# Commentary: Neural substrates of embodied natural beauty and social endowed beauty: An fMRI study

**DOI:** 10.3389/fnhum.2017.00596

**Published:** 2017-12-05

**Authors:** Marcos Nadal, Víctor Gallardo, Gisèle Marty

**Affiliations:** ^1^Department of Psychology, University of the Balearic Islands, Palma, Spain; ^2^Human Evolution and Cognition Group, Spanish National Research Council (CSIC), University of the Balearic Islands, Palma, Spain; ^3^Hospital Can Misses, Ibiza, Spain

**Keywords:** neuroaesthetics, beauty, objective, subjective, sense of beauty

The use of neuroimaging techniques to study aesthetic valuation has invigorated scientific aesthetics (Chatterjee, 2011; Nadal and Pearce, 2011). These techniques have improved our understanding of the relation between psychological processes involved in aesthetic valuation and the underlying neural mechanisms, they have made it possible to study cognitive or affective processes unaccompanied by overt behavioral responses, and they have provided crucial constraints on cognitive theories and models (Pearce et al., 2016). Not only have neuroimaging techniques led to new questions about aesthetics, they have produced new evidence capable of settling old debates.

In this vein, Zhang et al. (2017) recently used fMRI to explore the dispute between objectivist and subjectivist philosophies of beauty: Is beauty a quality of objects or a quality we attribute to objects? They asked participants to judge unfamiliar ancient Chinese characters as beautiful or ugly. Half of the characters were pictographs, referring to concrete objects and outlining their shape. The other half were ideographic symbols, referring to abstract social concepts. The authors assumed that beauty judgments of pictographs were based on their objective features, and beauty judgments of ideographs on their subjective socially constructed meanings.

Zhang et al. (2017) found widespread brain activity common to judgments of both sorts of characters, but they also found activity in certain brain regions specific either to judging the beauty of pictographs or judging the beauty of ideographs. They saw in these specific patterns the neural signatures of two distinct kinds of beauty, one related to object features and another to subjective processes. Zhang et al. (2017) argued that their results constitute evidence for a sense of beauty that responds to two different kinds of attributes: objective features (“embodied natural beauty”), and subjective social constructions (“social endowed beauty”).

However, motivated by their assumption that pictographs are judged for their objective features and ideographs for their subjective social meanings, Zhang et al. (2017) overlooked the most parsimonious interpretation of their results. Differences in brain activity related to the beauty judgments of pictographs and ideographs most probably owe to the former being representational and the latter being abstract. That the characters differed in abstraction is a matter of fact: they were chosen so. That they differed as to the source of their beauty is a matter of unsupported speculation: the objective features of ideographs and the meanings of pictographs can also be judged as beautiful or ugly. Zhang et al. (2017), thus, did not identify brain activity corresponding to “embodied natural beauty” and “social endowed beauty,” but brain activity corresponding to representational and abstract stimuli (Lengger et al., 2007; Fairhall and Ishai, 2008; Cattaneo et al., 2014, 2015, 2017).

Furthermore, Zhang et al.'s (2017) notion of a sense of beauty that responds to certain attributes is untenable given the abundant evidence showing, first, that there is no such thing as a sense of beauty and, second, that aesthetic valuation is not a response triggered by object features.

A century and a half of experimental research on art and aesthetics has yielded no trace of mental or neural processes particular to aesthetic valuation (Brown et al., 2011; Nadal, 2013; Chatterjee and Vartanian, 2014). The evidence actually shows that aesthetic valuation relies on the very same brain circuits involved in appraising the value of biologically relevant objects depending on one's state and goals (Skov, 2010; Brown et al., 2011; Salimpoor and Zatorre, 2013; Vartanian and Skov, 2014; Pearce et al., 2016; Mallik et al., 2017). These circuits compute the value of various sorts of objects and prospects, from the most basic and tangible, like food and sex, to the most abstract, like money and art (Levy and Glimcher, 2012; Ruff and Fehr, 2014; Berridge and Kringelbach, 2015). The notion of an aesthetic sense, faculty, or process is merely a vestige of Eighteenth century British Enlightenment (Kivy, 2003). It has no empirical support.

The conception of beauty as a response triggered by object features does not hold up to the evidence either. This conception is an expression of naïve realism, the composite belief that (1) properties such as color, form, or sound are attributes of objects in the world; (2) that perceiving is a stimuli-driven transformation of sensory input into coherent percepts; and (3) that the general function of cognition is to create accurate representations of the world (Neisser, 1967; Varela et al., 1991). Despite its intuitive appeal, naïve realism is refuted by the most basic facts of perception and cognition. First, color, form, and sound are not properties of objects, but attributes of our experience of objects. A perceived color, for instance, does not correspond with locally reflected light. Color constancy, simultaneous color contrast, and other phenomena demonstrate that “we cannot account for our experience of color as an attribute of things in the world by appealing simply to the intensity and wavelength composition of the light reflected from an area” (Varela et al., 1991, p. 160–161). Second, the brain is not a stimuli-driven system that reacts to external triggers, and perception is not a passive taking-in of stimuli (Neisser, 1967; Singer, 2013). Rather, the brain is a prediction-driven system that anticipates input, and perception is the active comparing of sensory features with predictions based on stored knowledge (Clark, 2013; Engel et al., 2013), past experience (Alink et al., 2010), global configuration (Murray et al., 2002), expectations (Egner et al., 2010), and context (Bar, 2004; Oliva and Torralba, 2007). Third, the function of cognition is not to produce an accurate representation of the world, but to bring meaning to it. We do so by interacting with the world based on what we know and believe about it, what we expect from it, and what we need and want from it (Bruner, 1990).

In sum, there is no such thing as a sense of beauty that responds to certain object attributes. If anything, beauty is an attribute of our experience of objects brought about by the activity of domain-general brain systems that seek to make meaning of those objects, their features, and their value to us, based on expectations and predictions (Salimpoor et al., 2011; Egermann et al., 2013), beliefs (Kirk et al., 2009b; Noguchi and Murota, 2013; Locher et al., 2015; Pelowski et al., 2017b), prior experience and expertise (Kirk et al., 2009a; Harvey et al., 2010; Pang et al., 2013), currently available information (Lengger et al., 2007; Swami, 2013), and context (Gartus and Leder, 2014; Brieber et al., 2015; Pelowski et al., 2017a).

## Author contributions

MN and VG conceived the research. MN, VG, and GM wrote the paper together.

### Conflict of interest statement

The authors declare that the research was conducted in the absence of any commercial or financial relationships that could be construed as a potential conflict of interest.
